# Early experience with the SHURUI single port serpentine arm robotic system with an auxiliary port in gastrointestinal surgery: a descriptive case series

**DOI:** 10.3389/fsurg.2026.1957028

**Published:** 2026-10-15

**Authors:** Dipesh Kumar Yadav, Yuncheng Luo, Zhangwei Yang, Yi Huang, Hanzhang Huang, Sisi Lin, Min Li, Dandan Bao, Yiren Hu

**Affiliations:** Department of General Surgery, Wenzhou People’s Hospital, The Third Clinical Institute Affiliated to Wenzhou Medical University, Wenzhou, China

**Keywords:** colectomy, gastrectomy, gastrointestinal malignancy, SHURUI system, single port robotic surgery

## Abstract

**Background:**

The SHURUI single-port (SP) robotic system is a novel platform with serpentine instruments designed for reduced-access surgery. Clinical experience in gastrointestinal malignancies remains limited. This exploratory, descriptive case series reports initial technical experience with the SHURUI SP system in a consecutive series of gastric and colorectal cancer resections.

**Methods:**

This retrospective case series included six consecutive patients who underwent SP robotic gastrointestinal cancer surgery between November 2025 and December 2025 at a single tertiary center. Procedures comprised rectal cancer resection with total mesorectal excision (*n* = 2), left hemicolectomy (*n* = 1), right hemicolectomy with D2 lymphadenectomy (*n* = 1), distal gastrectomy with D2 lymphadenectomy (*n* = 1), and total gastrectomy with extended D2 lymphadenectomy (*n* = 1). All cases used a single periumbilical incision (3–4 cm) and a 12 mm auxiliary port for energy devices and staplers. Outcomes included operative parameters, complications (Clavien-Dindo), pathological metrics, and hospital costs.

**Results:**

All six procedures were completed without conversion. Median operative time was 332.5 min (155–402); median estimated blood loss was 70 mL (10–200). No intraoperative complications or transfusions occurred. Postoperative Grade II complications occurred in two patients (33.3%): anastomotic bleeding requiring transfusion and wound infection. Grade I transient fever occurred in two additional patients (33.3%). No Grade III–V complications, reoperations, or 30-day mortality occurred. All resections achieved R0 margins; median lymph node yield was 18.5 (12–28). Median hospital stay was 14 days (12–20). Mean total hospital cost was ¥30,573 ± ¥14,725, exclusive of robotic platform and instrument fees, which were waived during this initial evaluation period.

**Conclusions:**

In this initial series, the SHURUI SP robotic system achieved complete resection without conversion in all six cases. R0 resection was obtained in every case with no severe early complications; however, the small sample size precludes definitive safety assessment, and oncologic adequacy requires long-term follow-up. The system proved technically feasible for complex gastric and colorectal cancer resections, though the absence of an integrated energy channel necessitated an auxiliary port in all cases, and operative times reflected an early learning curve. Larger comparative studies with long-term follow-up are needed to define the role of this platform.

## 1 Introduction

The evolution of minimally invasive surgery has progressed from multiport laparoscopy to multiport robotic surgery, and subsequently to reduced port and single port (SP) approaches (1, 2). SP laparoscopic surgery utilizes a single incision, typically concealed within the umbilicus, to accommodate all instruments, offering the advantages of reduced postoperative pain, fewer wound complications, and improved cosmetic outcomes (2–4). However, this approach is constrained by ergonomic limitations, including instrument collision, loss of triangulation, and crossed instrument manipulation (5).

SP robotic systems have addressed these limitations through three-dimensional visualization, tremor filtration, and restoration of triangulation despite the parallel port configuration (6). The da Vinci SP system (Intuitive Surgical, Sunnyvale, CA, USA) was launched in 2018 and has been successfully applied in a variety of abdominal procedures (6). Nevertheless, the substantial cost and restricted availability of this platform have prompted the development of alternative systems.

The SHURUI SP robotic surgical system (SR ENS 600; Beijing Surgerii Robotics Co., Ltd.) represents a novel platform specifically designed for SP procedures. In contrast to modified multiport systems, this platform incorporates four robotic arms within a single port through deformable serpentine instruments based on dual continuum mechanism technology. These instruments are constructed from nickel titanium alloy, providing multi-degree-of-freedom articulation and a wide range of motion within confined spaces (7, 8). The system received regulatory approval from the National Medical Products Administration (NMPA) in 2023 (8).

The mechanical design of the SHURUI platform differs meaningfully from that of the da Vinci SP system. The da Vinci SP employs rigid, individually articulated 6 mm instruments with two articulation points, whereas the SHURUI SP uses 8 mm serpentine instruments with a single articulation point (8). This design allows the instruments to curve around one another without rigid elbow joints, enabling them to reach around the operative field within the single port. The trade-off is greater flexibility but reduced rigidity compared with da Vinci SP instruments.

While the da Vinci SP system has been increasingly reported for colorectal and gastric cancer resection (6), clinical experience with the SHURUI platform in complex gastrointestinal malignancies remains limited. We herein report our initial experience with the SHURUI SP robotic system in gastrointestinal malignancies, assessing technical feasibility, surgical outcomes, and pathological metrics (margin status and lymph node yield), thereby contributing to the emerging evidence base for this platform.

## 2 Patients and methods

### 2.1 Patients

This retrospective study included six consecutive patients who underwent gastrointestinal surgery for malignant disease using the SHURUI SP robotic surgical system at Wenzhou People's Hospital between November 2025 and December 2025. The study was conducted in compliance with the principles of the Declaration of Helsinki (9) and received approval from the Institutional Review Board of Wenzhou People's Hospital. Written informed consent was obtained from all patients or their legal representatives. Reporting adheres to the STROBE guidelines (10).

Inclusion criteria for the SHURUI SP robotic surgery were: (1) age 18–80 years; (2) American Society of Anesthesiologists (ASA) physical status I–III (11); (3) body mass index <35 kg/m^2^ (a technical precaution, as the fixed single-port trocar geometry may increase instrument collision and reduce serpentine arm mobility in patients with a thick abdominal wall); (4) indication for major gastrointestinal resection for suspected or confirmed malignancy; (5) no evidence of distant metastases on preoperative imaging; and (6) informed consent for the SP robotic platform. Exclusion criteria were emergency surgery, previous major abdominal surgery, active intra-abdominal infection, severe cardiopulmonary disease, and bleeding disorders.

A standardized collection form was used to systematically extract data from the Electronic Medical Records (EMR). Preoperative variables included demographic information (sex, age, BMI, tumor location), clinical symptoms, and laboratory values. Intraoperative data captured operative time, estimated blood loss, and procedural details of the robotic surgery. Postoperative parameters recorded were laboratory values on postoperative day (POD) 1, time to first flatus, time to first liquid intake, complications (anastomotic leakage, bleeding, gastroparesis, chylous leakage, surgical site infection, pulmonary infection), postoperative hospital stay, total hospital stay, and total hospitalization costs. Pathological data consisted of tumor location, histological type, maximum tumor diameter, tumor differentiation, resection margin status, depth of invasion, pathological stage, total lymph nodes harvested, number of positive lymph nodes, and comprehensive pathological staging.

### 2.2 Preoperative workup and surgical planning

All patients underwent routine blood tests, serum tumor markers (CEA, CA19-9), nutritional assessment (albumin, prealbumin), and cardiopulmonary evaluation as indicated. Contrast-enhanced computed tomography (CT) and magnetic resonance imaging (MRI) were performed according to standard oncologic staging protocols. Preoperative colonoscopy or gastroscopy with endoscopic biopsy was performed to confirm histological diagnosis and precisely localize the tumor.

Comorbidities including hypertension and diabetes were optimized before surgery to ensure that patients met the required ASA physical status criteria. Procedure selection and the extent of lymphadenectomy were guided by tumor location and clinical stage, following established guidelines: for gastric malignancies, in accordance with the Japanese Gastric Cancer Treatment Guidelines 2018 (5th edition) (12); for colonic malignancies, following complete mesocolic excision (CME) principles with high ligation of feeding vessels (13); and for rectal malignancies, according to total mesorectal excision (TME) principles (14).

Achieving R0 resection was a core surgical objective. For gastric cancer, intraoperative determination of required gross resection margins depended on suspected T-stage: >2 cm for T1 tumors, ≥3 cm for T2 tumors with favorable morphology, and ≥5 cm for advanced T2 or T3–T4 tumors. When a 5 cm margin was anatomically unattainable (e.g., tumors involving the esophagogastric junction or pylorus), intraoperative frozen section analysis was used to confirm negative margins (15). For colonic cancer, a minimum 5 cm proximal and distal mural margin was required with central vascular ligation (13). For rectal cancer, TME was performed with a minimum distal margin of 1–2 cm, and circumferential resection margin (CRM) negativity was confirmed on postoperative pathological assessment (14).

### 2.3 Surgical platform and instrumentation

All procedures were performed using the SHURUI SP robotic surgical system. The technical features of the system have been described in detail elsewhere (7, 8). Briefly, the platform comprises a surgeon console and patient cart with four arms (three instrument arms and one camera arm) that pass through a single multichannel trocar. The serpentine instruments utilize dual-continuum mechanism technology with nickel-titanium alloy construction, providing multi-degree-of-freedom articulation. The system provides three-dimensional (3D) visualization through a 30° angled 8.5 mm robotic endoscope. Representative images of the surgeon console and patient cart are shown in Figure 1.

**Figure 1:**
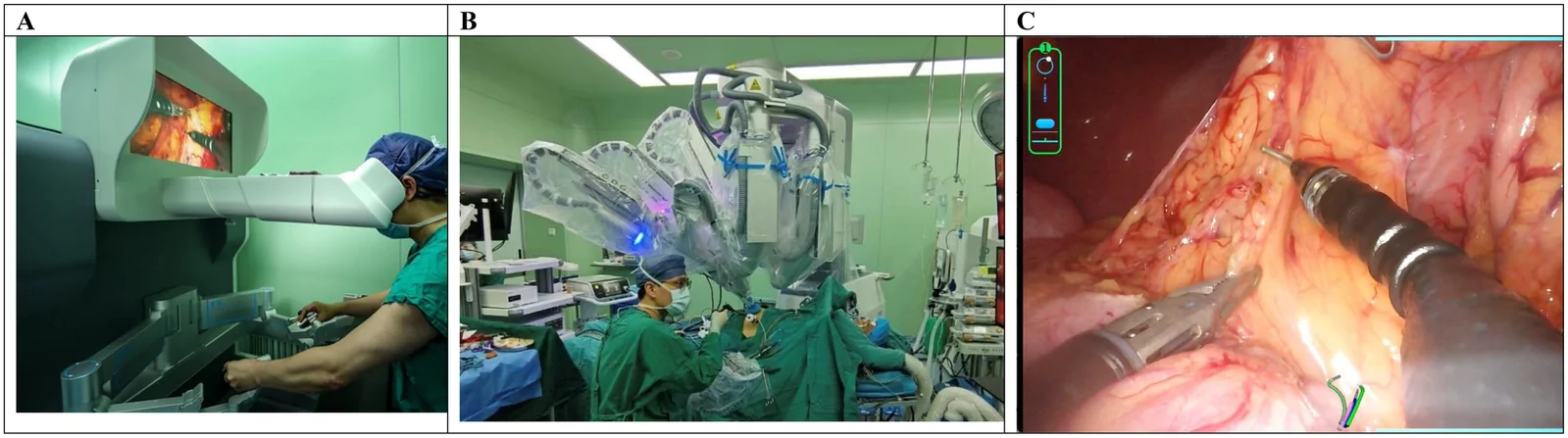
The SHURUI single-port robotic system. **(A)** Surgeon console. **(B)** Patient-side cart showing the single-port multichannel trocar and the auxiliary 12 mm port for assistance. **(C)** Intraoperative view during SHURUI single-port robotic gastric resection, showing serpentine forceps (left) and monopolar scissors (right) articulating through the single multichannel trocar.

### 2.4 Patient positioning

Following general anesthesia induction, patients were positioned according to procedure: modified lithotomy with steep Trendelenburg tilt for rectal resections, or supine with reverse Trendelenburg for colonic and gastric procedures.

### 2.5 Port placement and docking

A 3–4 cm vertical periumbilical incision was made, and pneumoperitoneum (12–15 mmHg) was established using the Hasson open technique. A multichannel single-port trocar was inserted through this incision. Because the SHURUI SP platform lacks a dedicated energy device channel, a 12 mm auxiliary port was routinely placed in the anterior axillary line (right side for gastric and left-sided colonic procedures; left side for right hemicolectomy) to enable assistant-controlled use of the harmonic scalpel, laparoscopic ultrasound (LUS), suction-irrigation, clip appliers, and staplers. The robot was positioned according to the specific procedure to allow optimal access to the operative field, with the assistant standing at the contralateral side of the patient.

Through the single-port trocar, the 3D robotic endoscope and three serpentine instruments were introduced: bipolar fenestrated grasping forceps (left hand), monopolar curved scissors (right hand), and atraumatic grasping forceps (third arm). The robotic arms were arranged circumferentially with the camera at the 12 o'clock position and instrument arms at 3, 6, and 9 o'clock positions. Docking was performed without arm collision, utilizing the platform's dual-continuum mechanism to maximize range of motion within the confined single-port space.

### 2.6 Surgical technique

All procedures were performed by a single surgical team experienced in multiport robotic and laparoscopic gastrointestinal surgery, although none had prior clinical experience with the SHURUI SP platform. Procedures comprised radical rectal resection with total mesorectal excision (*n* = 2), left hemicolectomy (*n* = 1), right hemicolectomy (*n* = 1), distal gastrectomy (*n* = 1), and total gastrectomy (*n* = 1).

#### 2.6.1 Intraoperative tumor localization

Intraoperative LUS was introduced through the 12 mm auxiliary port for tumor localization in all cases (15). After confirmation by LUS, tumor margins were marked with vascular clips under robotic vision. Intraoperative gastroscopy or colonoscopy was reserved as a salvage technique for cases in which LUS localization was equivocal.

#### 2.6.2 Colorectal procedures

##### 2.6.2.1 Right hemicolectomy

Using a medial-to-lateral approach, the ileocolic pedicle was elevated and the peritoneum was incised at its base. The ileocolic vessels were skeletonized at their origin from the superior mesenteric axis, clipped, and divided. D2 lymphadenectomy was completed along the ileocolic, right colic, and right branch of the middle colic vessels. The right colon was mobilized anterior to Toldt's fascia, with preservation of the right ureter and gonadal vessels and protection of the duodenum and pancreatic head. The transverse colon and terminal ileum were divided using an endoscopic linear stapler (60 mm reload) introduced through the auxiliary port.

##### 2.6.2.2 Left hemicolectomy

Using a medial-to-lateral approach, the sigmoid colon was retracted to expose the sacral promontory. The peritoneum was incised at the base of the inferior mesenteric artery (IMA). After D2 lymphadenectomy (stations 241 and 242), the IMA and inferior mesenteric vein (IMV) were divided at their origins. The left colon was mobilized in the retroperitoneal plane, preserving the left ureter and gonadal vessels, with splenic flexure takedown performed as required.

##### 2.6.2.3 Rectal cancer resection with total mesorectal excision

Following high ligation of the IMA and IMV at the inferior border of the pancreas, the retrorectal plane was entered posteriorly at the sacral promontory. Sharp dissection proceeded in the avascular plane between the mesorectal fascia and the presacral fascia, preserving the hypogastric nerves and pelvic autonomic plexuses. Lateral dissection identified and preserved the pelvic splanchnic nerves. Anterior dissection separated the rectum from the prostate (in males) or the vagina (in females) in the plane between Denonvilliers’ fascia and the anterior mesorectal fascia. The rectum was divided using a laparoscopic linear stapler introduced through the auxiliary port.

#### 2.6.3 Gastric procedures

##### 2.6.3.1 Distal gastrectomy with D2 lymphadenectomy

The greater omentum was resected along the gastroepiploic arcade, and the right gastroepiploic vessels were ligated at their origins. D2 lymphadenectomy (stations 1–6, 7, 8a, 9, 11p, and 12a) was performed, followed by division of the left gastric artery at its origin. The duodenum was transected 2 cm distal to the pylorus using an endoscopic linear stapler, and the proximal stomach was transected at the appropriate level.

##### 2.6.3.2 Total gastrectomy with extended D2 lymphadenectomy

Extended dissection included stations 8a, 9, 11p, 11d, 19, and 20. The esophagus was mobilized circumferentially, the phrenoesophageal ligament was divided, and the distal esophagus was transected with an endoscopic linear stapler.

#### 2.6.4 Specimen extraction and reconstruction

After completion of the resection, the periumbilical incision was extended superiorly to 5–6 cm. A wound protector was inserted, and the specimen was extracted through the same incision.

##### 2.6.4.1 Colorectal reconstruction

For right hemicolectomy, an extracorporeal side-to-side ileocolic anastomosis was performed. For left hemicolectomy, a colocolic anastomosis was done. For rectal resections, an end-to-end colorectal anastomosis was completed using a transanal circular stapler (29 mm diameter).

##### 2.6.4.2 Gastric reconstruction

Roux-en-Y reconstruction was performed extracorporeally. The jejunum was divided 20 cm distal to the ligament of Treitz. For distal gastrectomy, a side-to-side stapled gastrojejunostomy was done. For total gastrectomy, an esophagojejunostomy was performed using a circular stapler (25 mm diameter), followed by a jejunojejunostomy 40–50 cm distal to the primary anastomosis.

#### 2.6.5 Drainage and closure

A closed-suction drain was placed in all cases, positioned appropriately according to the procedure and introduced through the auxiliary port site. The fascial layer was closed with absorbable sutures, and the skin was approximated with subcuticular sutures. Figure 2 shows the postoperative incisions. All procedures were performed by a single surgical team specialized in minimally invasive gastrointestinal surgery, and no conversion to open or multiport laparoscopy was required.

**Figure 2:**
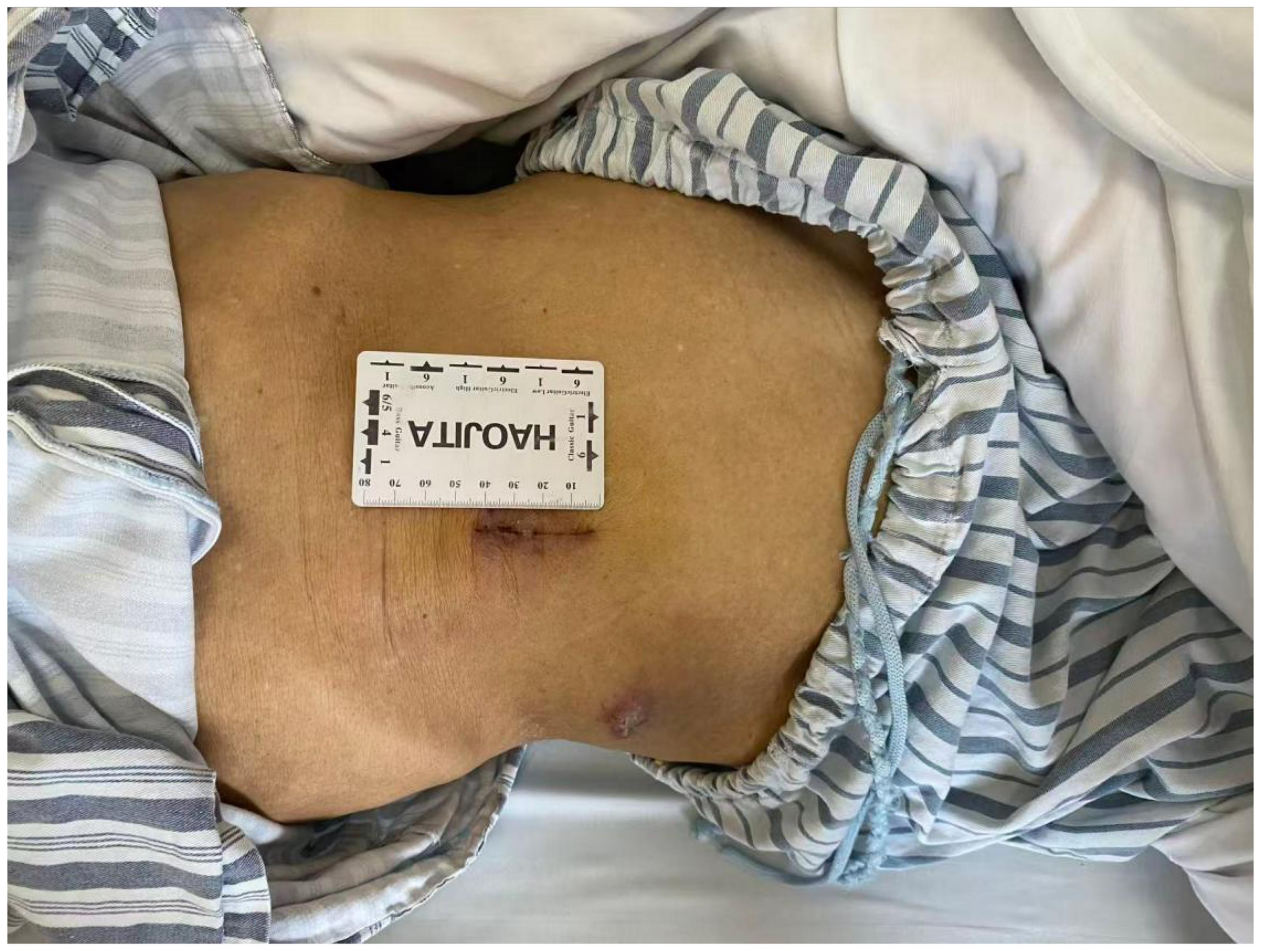
Postoperative incisions. The main periumbilical incision measures approximately 4.5 cm. The auxiliary port site is visible as a small mark in the anterior axillary line.

### 2.7 Postoperative management

All patients were managed according to the Enhanced Recovery After Surgery (ERAS) protocol (15, 16), which included early mobilization, initiation of clear oral liquids after return of bowel function, multimodal analgesia, and routine antiemesis.

### 2.8 Outcome measures

Primary outcomes were technical feasibility (completion without conversion), total operative time, estimated blood loss, and intraoperative complications. Secondary outcomes comprised postoperative pain scores (0–10 visual analog scale) (17), time to first flatus, time to resumption of oral intake, length of hospital stay, postoperative complications (graded according to the Clavien-Dindo classification) (18), resection margin status, lymph node yield, and total hospital costs.

### 2.9 Statistical analysis

Given the case series design and small sample size (*n* = 6), descriptive statistics were employed. Continuous variables are reported as median with range or mean ± standard deviation. Categorical variables are reported as frequencies and percentages. No inferential statistical testing was performed. All results are reported descriptively; no *p*-values or between-group comparisons are presented.

## 3 Results

### 3.1 Patient demographics

The cohort comprised three men and three women, with a median age of 68.5 years (45–72 years). Mean BMI was 24.0 ± 4.1 kg/m^2^. ASA class was II in four patients (66.7%) and III in two patients (33.3%). Tumor locations were the rectum (*n* = 2), sigmoid colon (*n* = 1), cecum (*n* = 1), distal stomach (*n* = 1), and gastric cardia (*n* = 1). Detailed baseline characteristics are presented in Table 1.

**Table 1:** Patient demographics and preoperative characteristics.

Characteristic	Value
Age, years, median (range)	68.5 (45–72)
Gender, *n* (%)	
Male	3 (50.0)
Female	3 (50.0)
BMI, kg/m^2^, mean ± SD (range)	24.0 ± 4.1 (17.6–28.9)
ASA classification, *n* (%)	
II	4 (66.7)
III	2 (33.3)
Previous abdominal surgery, *n* (%)	0 (0)
Preoperative albumin, g/L, mean ± SD	37.5 ± 3.2
Preoperative hemoglobin, g/L, mean ± SD	120.7 ± 21.4
Preoperative CRP, mg/L, median (range)	3.0 (1.0–15.0)
Tumor location, *n* (%)	
Rectum	2 (33.3)
Sigmoid colon	1 (16.7)
Cecum	1 (16.7)
Distal stomach	1 (16.7)
Gastric cardia	1 (16.7)

### 3.2 Operative outcomes

All six procedures were completed without conversion to open surgery or multiport laparoscopy. Median total operative time was 332.5 min (155–402 min). The shortest procedure was left hemicolectomy (155 min), and the longest was rectal resection (402 min). Median estimated blood loss was 70 mL (10–200 mL). No patient required intraoperative blood transfusion, and no intraoperative complications occurred. Table 2 summarizes intraoperative outcomes.

**Table 2:** Intraoperative outcomes.

Case	Procedure	Total op time (min)	Docking time (min)	Console time (min)	Blood loss (mL)	Auxiliary port	Conversion
1	Rectal resection	402	29	282	50	Yes	No
2	Total gastrectomy	400	29	238	90	Yes	No
3	Distal gastrectomy	352	16	210	200	Yes	No
4	Right hemicolectomy	313	28	252	70	Yes	No
5	Rectal resection	270	29	156	70	Yes	No
6	Left hemicolectomy	155	16	51	10	Yes	No

### 3.3 Pathological outcomes

All resections achieved R0 margins. The median number of lymph nodes harvested was 18.5 (12–28 nodes). Lymph node metastases were present in three patients (Cases 1, 3, and 4). Lymphovascular invasion was observed in four patients, and perineural invasion in three. Pathological staging was done according to AJCC 8th edition. Individual patient data are presented in Table 3.

**Table 3:** Pathological outcomes.

Case	Tumor location	Pathological stage (AJCC 8th)	Lymph nodes harvested	Positive nodes	Lymphovascular invasion	Perineural invasion	Resection margin
1	Rectum	pT3N2bM0 (IIIC)	28	18	Yes	Yes	R0
2	Gastric cardia	pT3N0M0 (IIA)	27	0	Yes	Yes	R0
3	Distal stomach	pT2N3aM0 (IIIA)	22	8	Yes	Yes	R0
4	Cecum	pT3N1M0 (IIIB)	15	2	Yes	No	R0
5	Rectum	pT2N0M0 (I)	14	0	No	No	R0
6	Sigmoid colon	pT1N0M0 (I)	12	0	No	No	R0

### 3.4 Postoperative outcomes and hospital costs

The median time to first flatus was 33.5 h (14–75 h). Median postoperative hospital stay was 14 days (12–20 days). Gastric procedures (distal and total gastrectomy) had a median stay of 18.5 days (17–20 days), whereas colorectal procedures had a median stay of 13.5 days (12–14 days).

Postoperative complications occurred in four patients (66.7%). Grade I complications (transient fever) occurred in two patients (Cases 1 and 2; 33.3%). Grade II complications occurred in two patients (33.3%): anastomotic bleeding requiring transfusion (Case 4) and wound infection (Case 3). Both were managed conservatively without reoperation. No grade III–V complications, reoperations, or 30-day mortality occurred. When Grade I events were excluded, the rate of clinically significant (Grade II) morbidity was 33.3%.

The median pain score at 24 h was 2.5 (2–3). The highest score (3) occurred in Cases 2, 3, and 6. Preoperative C-reactive protein (CRP) levels were normal in most patients, with a median of 3.0 mg/L (1.0–15.0 mg/L). On POD 1, CRP increased to a median of 48.6 mg/L (36.2–80.3 mg/L). The highest postoperative CRP (80.3 mg/L) occurred in the patient who underwent total gastrectomy (Case 2), who also had the longest hospital stay (20 days) and a grade I fever.

The mean total hospital cost was ¥30,573 ± ¥14,725 (17,566–54,460). The highest cost was for total gastrectomy (Case 2, ¥54,460), and the lowest was for right hemicolectomy (Case 4, ¥17,566). It is important to note that these figures represent hospitalization costs only and do not include robotic platform usage fees or instrument costs, as the SHURUI system was provided at no charge during this initial evaluation period. Postoperative outcomes are summarized in Table 4.

**Table 4:** Postoperative outcomes.

Case	Procedure	Time to first flatus (h)	Liquid diet (POD)	Complications (clavien-dindo)	Hospital stay (days)	Pain score VAS 24 h	Postop day 1 CRP (mg/L)	Hospital cost ¥
1	Rectal resection	67	5	Grade I (fever)	14	2	45.9	19,906
2	Total gastrectomy	15	5	Grade I (fever)	20	3	80.3	54,460
3	Distal gastrectomy	14	7	Grade II (wound infection)	17	3	57.6	42,897
4	Right hemicolectomy	14	4	Grade II (anastomotic bleeding)	13	2	36.2	17,566
5	Rectal resection	52	6	None	14	2	44.9	24,395
6	Left hemicolectomy	75	5	None	12	3	51.3	24,212

POD, postoperative day; VAS, visual analog scale; CRP, C-reactive protein.

## 4 Discussion

This case series represents our earlier clinical experiences with the SHURUI SP robotic system for complex gastrointestinal malignancies. Our findings demonstrate that SHURUI SP robotic platform may enables technically complete resections without conversion for gastric and colorectal procedures. However, the operative times, lymph node yields, and specific mechanical characteristics observed reflect both the inherent constraints of early-generation single-port technology and the learning curve associated with serpentine instrument control.

The 100% completion rate without conversion aligns with previous reports of SP robotic surgery for gastric (19) and colon (20) malignancies. However, a systematic review of SP robotic colorectal surgery reported a pooled conversion rate of 0.72% (21), so conversion-free outcomes in small samples require cautious interpretation. The absence of intraoperative complications or transfusions in our series is reassuring but cannot establish an acceptable safety profile given the small sample size.

However, the median total operative time of 332.5 min (155–402 min) requires contextual interpretation. A recent propensity-matched comparison of da Vinci SP vs. multi-port da Vinci™ Xi system distal gastrectomy reported median operative times of 329 vs. 414 min, suggesting experienced SP operators can match or exceed multiport efficiency (22). Our median time of 332.5 min falls within this range, though our wide temporal spread (155–402 min) reflects the learning curve. A meta-analysis of robotic D2 gastrectomy identified 25–50 cases as the proficiency threshold, with a 35% (94.6 min) time reduction after mastery (23). Our six-case experience clearly resides within the initial learning phase, explaining the observed variability.

The median estimated blood loss of 70 mL (10–200), comparing favorably with SP colorectal series (33–103 mL) (24) and a da Vinci SP gastrectomy trial (50 mL, 9–150) (19). The upper range value of 200 mL in our series occurred in the distal gastrectomy case presenting with a bleeding ulcer, where preoperative hemorrhage and coagulopathy contributed to intraoperative blood loss. Excluding this outlier, the median blood loss for the remaining five cases was substantially lower. The median docking time of 28.5 min was comparable to early SP colectomy reports (14–24 min) (25).

All resections were R0, median lymph nodes 18.5, meeting gastric (≥15) and colorectal (≥12) thresholds (26, 27). However, the lymph node yields in our series appear modest compared with high-volume SP and multiport robotic series. A propensity-matched study of SP vs. multiport da Vinci distal gastrectomy reported mean 46 vs. 32 lymph node retrieval (22); while a Korean multi-institutional study found 33, 47, and 30 nodes for multiport, single-site, and SP gastrectomy, respectively (28). Our gastric resections yielded 22 and 27 nodes, meeting D2 requirements but falling below these benchmarks. This discrepancy should not be attributed to an inherent limitation of the SHURUI platform. Rather, it likely reflects the small sample size, heterogeneous case mix, patient selection, and the early learning curve of the operating team, for whom lymphadenectomy with serpentine instruments may be steeper than with rigid robotic arms. These observations suggest that the SP approach with serpentine instruments may be particularly advantageous for benign or premalignant lesions, where lymphadenectomy requirements are lower and the benefits of reduced abdominal wall trauma may be more pronounced. For advanced cancers requiring D2 dissection, careful patient selection is advisable until proficiency improves.

Clinically significant (Grade II) complications occurred in two patients (33.3%). This rate exceeds the 8.3%–16.7% reported for da Vinci SP gastrectomy and colorectal procedures (22, 29), but our mix cases limits direct comparison. The absence of major complications argues against platform-specific safety concerns. We did, however, observe higher-than-expected periumbilical port-site inflammation, characterized by fat necrosis and tissue induration without infection. This appears attributable to mechanical stress from continuous manipulation of the serpentine arms through the single multichannel trocar, whose back-and-forth movement generates friction and pressure against the fascial and subcutaneous tissues that may exceed that of rigid multiport instruments. This suggests that the single-incision approach with serpentine technology may incur a distinct tissue trauma profile. To minimize this, we recommend intermittent instrument retraction, gentle and deliberate arm manipulation, avoidance of excessive lateral torque, periodic inspection of the periumbilical skin during long procedures, and lubrication of the trocar–instrument interface. These empirical measures warrant formal prospective evaluation.

The median hospital stay of 14 days (12–20) substantially exceeds fast-track benchmarks reported in earlier SP robotic surgeries studies for colorectal (20) and gastric (19, 22) surgeries. Our prolonged stays likely reflect institutional discharge protocols, cultural monitoring practices, and conservative discharge criteria rather than surgical morbidity *per se*. Gastric procedures demonstrated longer stays (median 18.5 days) compared with colorectal procedures (median 13.5 days), consistent with the greater physiological impact of gastrectomy and the higher complication rate observed in these cases.

The median time to first flatus of 33.5 h and low pain scores (median 2.5 on VAS) suggest adequate postoperative recovery and align with reported benefits of single-port approaches in reducing postoperative pain and accelerating gastrointestinal recovery (29). The CRP elevation patterns on postoperative day 1 (median 48.6 mg/L) were consistent with expected surgical stress responses (30).

The mean total hospital cost of ¥30,573 provides preliminary economic data for this platform, but must be interpreted with extreme caution. Robotic platform usage fees and instrument charges were entirely waived during this initial evaluation period. The reported mean cost therefore reflects only routine hospitalization expenses and substantially underestimates the true per-case economic burden. Accordingly, these data cannot be directly compared with published cost analyses of established robotic platforms, such as da Vinci or other multiport systems, in which instrument depreciation and usage fees are included—costs that can constitute a substantial proportion of total procedural expenses. A formal cost-effectiveness analysis incorporating all platform-related expenses, including acquisition, maintenance, disposable instruments, and operative time-dependent overheads, is required before any economic conclusions can be drawn, particularly given the SHURUI system's positioning as a potentially cost-accessible alternative to established platforms. Until such data are available, the cost figures reported here should be considered exploratory only.

Consistent with the reduced rigidity of the serpentine design described earlier, our experience revealed several mechanical characteristics that differ meaningfully from multiport robotic and conventional laparoscopic platforms. When using monopolar scissors to transect tissue under tension (e.g., mesenteric division or gastrosplenic ligament transection), the arm transmits visible tremor to the surgical field, forcing the console surgeon to reduce traction and stabilize carefully to avoid injuring adjacent vessels or organs. The nitinol backbone provides excellent articulation but lacks the damping of rigid robotic arms under reactive forces from high-tension dissection.

The coaxial arrangement of the camera and instrument arms within a single multichannel port introduces additional constraints. Unlike multiport configurations with independent entry points, the SHURUI system makes the entire surgical field pivot as a single unit on the abdominal wall entrance. This reduces external arm collisions but narrows the effective field of view compared with multiport robotic or laparoscopic surgery. Movement of the serpentine arms can also temporarily block the endoscopic view, requiring frequent scope repositioning that increases operative time and cognitive load. The 30-degree angled scope provides some compensation, but the need for repeated orientation adjustment during instrument manipulation represents a distinct disadvantage compared with multiport systems, where the camera remains stable regardless of instrument movement.

The absence of an integrated energy device channel—shared with the da Vinci SP platform—necessitated routine placement of a 12 mm auxiliary port in all cases. This requirement partially compromises the “single-incision” concept and fundamentally alters procedural workflow, as the bedside assistant must coordinate energy device activation, suction, and stapler deployment while avoiding collision with the serpentine arms occupying the central port. This limitation is common to single-port systems, including the da Vinci SP, which similarly requires specialized instrumentation or an assistant port for energy devices; both face greater instrumentation constraints than multiport platforms. The technical demands on assistants are substantial, as the serpentine arms’ wide range of motion within the single port creates a dynamic spatial envelope that assistant instruments must navigate without interference.

### 4.1 Limitations

This study has several limitations: it is retrospective, from a single center, with only six patients. The small sample size and single-center design limit generalizability and prevent meaningful statistical analysis. The case mix is heterogeneous, which shows the platform's versatility but makes procedure-specific conclusions difficult. We did not collect patient-reported outcomes or cosmetic satisfaction scores—both relevant endpoints given the port site inflammation we observed. Follow-up was short and limited to the immediate postoperative period; we cannot report disease-free survival, overall survival, local recurrence, or incisional hernia rates. Long-term oncologic follow-up is essential before the oncologic adequacy of this platform can be assessed. Finally, we had no control group, so we cannot judge noninferiority to established robotic platforms.

An additional important limitation concerns the hospital cost data. The reported costs were derived from EMR billing records and did not include robotic system usage fees, instrument charges, or platform depreciation costs, as the SHURUI system was provided at no charge during this initial evaluation period. These figures therefore substantially underestimate the true per-case economic burden and cannot be directly compared with cost data from established robotic platforms where such fees are incorporated. A formal cost-effectiveness analysis incorporating all platform-related expenses is required before any conclusions regarding economic viability can be drawn.

## 5 Conclusion

In this early experience, the SHURUI single-port robotic system with an auxiliary port allowed complete resection of gastric and colorectal cancers without conversion in all six cases. The serpentine instrument design offers better maneuverability through a single port but carries specific trade-offs: tremor during high-tension dissection, a narrower field of view from coaxial camera-arm integration, and the need for an auxiliary port due to the absence of an integrated energy channel. No severe early complications occurred, but the sample is too small to establish safety. R0 resection was achieved in all cases; however, oncologic adequacy cannot be assessed without long-term follow-up. Cost data were incomplete because platform and instrument fees were waived, and formal cost-effectiveness analysis is needed. Prospective comparative studies with long-term follow-up are required to define the optimal role of this cost-accessible platform in gastrointestinal oncology.

## Data Availability

The datasets presented in this study can be found in online repositories. The names of the repository/repositories and accession number(s) can be found in the article.
